# 应用凝血复衡策略治疗热射病性凝血病的经验总结

**DOI:** 10.3760/cma.j.cn121090-20241211-00557

**Published:** 2024-12

**Authors:** 青伟 林, 星平 邓, 慧强 刘, 诗凯 余, 景春 宋

**Affiliations:** 解放军联勤保障部队第九〇八医院重症医学科/南昌市血栓与止血学重点实验室，南昌 330002 Department of Critical Care Medicine, the 908th Hospital of Joint Logistics Support Forces of Chinese PLA, Nanchang 330002, China

## Abstract

报告1例20岁消防员特重型劳力型热射病性凝血病的救治经过。通过“五早一防”综合治疗策略，包括早降温、早抗凝、早补凝、早抗炎、早血液净化和预防并发症，成功救治了该患者。针对热射病性凝血病的处理，依据凝血复衡策略，即抗凝联合目标导向性替代治疗，联合血浆透析滤过（plasma diafiltration, PDF）治疗，有效改善患者凝血功能，且未发生出血及血栓事件。患者最终于入院第4天凝血功能基本恢复正常，第6天拔除气管插管，11 d转普通病房，21 d康复出院。

## Abstract (en)

This case report describes the treatment process of a severe exertional heat stroke-induced coagulopathy in a 20-year-old firefighter. Through a comprehensive treatment strategy of five early and one prevention, including early cooling, early anticoagulation, early coagulation factor supplementation, early anti-inflammation, early blood purification, and prevention of complications, the patient was successfully treated. For the management of heat stroke-induced coagulopathy, based on the coagulation rebalancing strategy-namely anticoagulation combined with goal-directed replacement therapy, along with plasma diafiltration（PDF）treatment-the patient's coagulation function was effectively improved without any bleeding or thrombotic events. The patient's coagulation function returned to normal by day 4 of hospitalization, the tracheal tube was removed on day 6, transferred to the general ward after 11 days, and was discharged after 21 days of rehabilitation.

热射病是可危及生命的热损伤疾病，常发生于高温、高湿环境下，尤其是在剧烈运动或职业暴露期间[1]。该病可迅速进展为多器官功能障碍综合征，其中凝血紊乱是常见的预后不良的独立危险因素[2]。热射病性凝血病的发病机制复杂，主要涉及炎症反应、内皮细胞损伤和凝血系统的异常[3]。首先，高热会引发全身性炎症反应，促使炎症因子（如细胞因子和趋化因子）大量释放，进一步激活凝血系统。其次，炎症反应导致内皮细胞损伤，增加血管通透性，促进血小板聚集和微血栓形成。若不进行干预，随着凝血因子的消耗和纤维蛋白降解产物的增加，微血栓广泛形成而致弥散性血管内凝血（disseminated intravascular coagulation, DIC），导致出血倾向和多器官功能障碍[4]。目前，热射病性凝血病的发病机制、早期诊断和精准治疗仍是临床医学研究的重点和难点。本病例报告旨在应用凝血复衡策略联合治疗1例特重型热射病性凝血病，阐述其临床特征及救治策略，为临床医师提供诊疗参考。

## 病例资料

患者系20岁消防员，于2021年7月2日16时左右（温度30 °C，湿度70％）开始进行10 km体能训练，17时左右跑至9 km处突然倒地，出现谵妄症状。急送入当地医院内科住院。入院测腋温达41.2 °C，诊断中暑，给予物理降温，补液等处理，降温效果不佳，且意识障碍进行性加重，血压进行性下降。于21时转入重症医学科，相关检验结果示白细胞计数22.08×10^9^/L，血红蛋白156 g/L，血小板计数192×10^9^/L，肌酐216 µmol/L，肌酸激酶391 U/L，肌酸同工酶32.3 U/L，乳酸脱氢酶447.5 U/L，肌红蛋白1 462 ng/ml，淀粉酶339.3 U/L，凝血实验显示凝血酶原时间（prothrombin time, PT）11.5 s，部分活化凝血酶原时间（activated partial thromboplastin time, APTT）21.4 s，纤维蛋白原1.62 g/L，凝血酶时间（thrombin time, TT）19.2 s，D-二聚体3.4 µg/ml。给予气管插管接呼吸机辅助呼吸，补液扩容，并行床旁血液净化治疗。因循环不稳定，去甲肾上腺素剂量上调至1 µg·kg^−1^·min^−1^，乳酸进行性升高至4.6 mmol/L，鉴于病情危重，于7月3日凌晨4时转入我院重症医学科进一步治疗。

入科查体：神志浅昏迷，体温38.3 °C，心率100次/min，血压90/46 mmHg（1 mmHg=0.133 kPa）（去甲肾上腺素2 µg·kg^−1^·min^−1^），气管插管接呼吸机辅助呼吸（氧浓度40％），双侧瞳孔等大等圆，直接1.5 mm，对光反射迟钝。背部及前胸部大面积瘀斑。大便失禁，约2 000 ml。入科检验：白细胞计数8.9×10^9^/L，中性粒细胞百分比85％，血红蛋白120 g/L，血小板计数59×10^9^/L；凝血功能：PT 27.6 s，APTT 38.4 s，纤维蛋白原1.08 g/L，TT 24.2 s，D-二聚体5.8 µg/ml，纤维蛋白降解产物（FDP）17.8 µg/ml、血栓调节蛋白（thrombomodulin, TM）12.4 TU/ml，凝血酶-抗凝血酶复合物（thrombin-antithrombin complex, TAT）33.9 ng/ml，纤溶酶-α2纤溶酶抑制剂复合物（plasmin-antiplasmin complex, PIC）4.18 µg/ml，组织型纤溶酶原激活物-抑制剂-1复合物（tissue plasminogen activator-plasminogen activator inhibitor-1 complex, t-PAIC）35.6 ng/ml；血栓弹力图普通杯凝血时间（R）为6.5 min，血块形成速率（K）7.0 min，血块形成动力学（α角）31.3°，血块最大强度（maximum amplitude, MA）36.5 mm，凝血指数−7.8（图1A）。肝肾功能：谷丙转氨酶50.3 U/L、谷草转氨酶75.7 U/L、总胆红素23 µmol/L，白蛋白25.4 g/L，肌酐211 µmol/L，尿素氮12.9 mmol/L；心肌酶谱：乳酸脱氢酶398 U/L，肌酸激酶1 524 U/L，肌酸酶同工酶39 U/L，心肌钙蛋白0.6 ng/ml，肌红蛋白978 µg/L；N末端B型利钠肽原（NT-proBNP）1 353 pg/ml，降钙素原（Procalcitonin, PCT）8.23 ng/ml；血气分析：pH 7.41，PCO_2_ 27 mmHg，PO_2_ 192 mmHg（氧合指数480 mmHg），Lac 4.0 mmol/L。急性生理与慢性健康评分Ⅱ（Acute Physiology and Chronic Health Evaluation, APACHE Ⅱ）21分，中国弥散性血管内凝血诊断积分系统（Chinese DIC scoring system，CDSS）[5]为8分。有创血流动力学监测示心输出指数（cardiac output index, CI）8.84 L·min^−1^·m^−2^，全心舒张末期容量指数（global end⁃diastolic volume, GEDI）844 ml/m^2^，系统血管阻力指数（systemic vascular resistance index, SVRI）438 dyn·s·cm^−5^·m^2^，血管外肺水指数（extravascular lung water, ELWI）6 ml/kg（表1）。入院诊断：劳力型热射病、多器官功能障碍综合征、热射病性凝血病、DIC、分布型休克、胃肠功能衰竭、低蛋白血症。

**图1:**
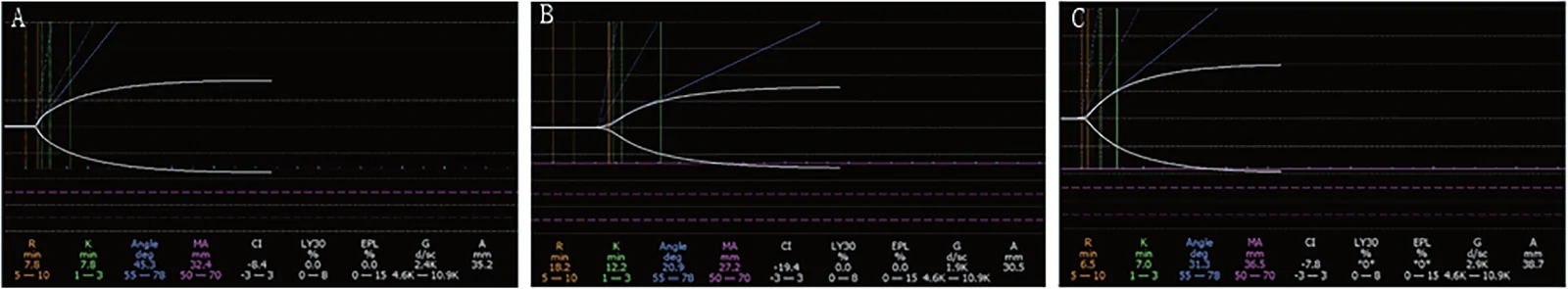
特重型劳力型热射病性凝血病患者血栓弹力图 **A** 血滤前普通杯；**B** 血滤6 h普通杯；**C** 血滤6 h肝素酶杯

**表1:** 特重型劳力型热射病性凝血病患者入科时及入科27 h有创血流动力学情况

指标名称	入科时	入科27 h	正常值参考范围
CI（L·min^−1^·m^−2^）	8.84	3.74	3～5
GEDI（ml/m^2^）	844	762	680～800
SVRI（dyn·s·cm^−5^·m^2^）	438	2074	1 700～2 400
ELWI（ml/kg）	6	7	3～7

**注** CI：心输出指数；GEDI：全心舒张末期容量指数；SVRI：系统血管阻力指数；ELWI：血管外肺水指数

治疗方案主要采用“五早一防”策略。①早降温：入科后体温波动在38.3 °C～39.2 °C之间，体温处于不稳定状态，严格按照热射病医院温度目标管理的方案进行将患者核心温度控制在38.0 °C以下，通过降温毯进行体外降温，并行连续肾脏替代疗法（Continuous Renal Replacement Therapy, CRRT）清除炎症介质的同时降低血温，采用监测肛温的方法持续监测核心温度的变化。②早补凝和抗凝：入科后根据血常规及凝血功能检验，CDSS评分达8分，血栓弹力图显示为低纤维蛋白原及血小板减少性低凝状态，当日给予输注人纤维蛋白原4 g，血小板1个治疗量、冷沉淀20单位，普通肝素2 mg/h泵入，根据血栓弹力图肝素酶对比实验滴定调节抗凝剂量维持在1～4 mg/h（图1B、C），在补凝抗凝的过程中2～3 d行双下肢血管超声检查1次，始终未发现血栓的形成。③CRRT治疗：患者入科后体温升高，浓茶样尿，肌红蛋白最高升至978 µg/L，肌酸激酶最高升至23 024 U/L，为降低血温，清除体内毒素，纠正凝血功能紊乱，行CRRT治疗，采用的模式主要有连续性静脉-静脉血液滤过透析（continuous veno-venous hemodiafiltration, CVVHDF）和血浆透析滤过（plasma diafiltration, PDF），共做PDF 3次（血浆共使用6 000 ml，第1次血浆用量1 600 ml，第2次血浆用量2 400 ml，第3次血浆用量2 000 ml），CVVHDF 5次，CRRT治疗4 d后尿量恢复至2 200 ml/d，茶色尿转清亮，肌红蛋白、肌酸激酶等各项指标逐渐恢复正常。④抗感染：入科时PCT高达8.23 ng/ml，结合入科时严重腹泻，考虑胃肠功能损伤后肠源性感染，留取血、尿、痰、粪便培养后，使用美罗培南联合万古霉素抗感染治疗。⑤容量管控：在有创血流动力学指导下严格进行容量管控，早期显示高心排低血管阻力型休克，前27 h积极补液（总入量13 420 ml，总出量5 330 ml），高排低阻改善（表1），血管活性药物下调。⑥其他治疗：镇痛镇静，肠内营养支持等对症治疗。经上述综合治疗后，于入院第4天凝血功能基本纠正至正常，第6天拔除气管插管，ICU住院11 d转普通病房。住院21 d康复出院。

## 讨论及文献复习

本案例为特重型劳力型热射病致多器官功能障碍综合征的典型案例。严重的凝血功能障碍是本病例的突出表现，发病早期即出现热射病性凝血病，在不到10 h进展为热射病性DIC。热射病性DIC属于血栓型DIC，需要进行积极的抗凝治疗。热射病性凝血病的治疗主要治疗原则为“五早一控”，即早降温、早抗凝、早补凝、早抗炎、早血液净化及控制并发症[3]。以下主要针对热射病性凝血病的诊断、基于抗凝及补凝原则制定的凝血复衡策略以及PDF对于凝血病的治疗意义进行讨论。

根据热射病性凝血病的诊断标准，患者发病后第一个凝血结果即可诊断为热射病性凝血病[6]，此时血小板计数仍保持在192×10^9^/L，这可能是热射病早期严重脱水导致血液浓缩，而造成血小板计数相对升高的结果。在经8 h后，血小板计数下降至59×10^9^/L，一方面有经补液后血液稀释的影响，另一方面是热射病性凝血病进一步发展至DIC的结果，CDSS评分达8分，同时凝血新四项的TAT 33.9 ng/ml提示凝血酶活动亢进，t-PAIC 35.6 ng/ml提示内皮损害。血栓弹力图结果提示整体凝血呈低凝状态，这与预后不良相关。从本例看，热射病性凝血病发展迅速，必须尽早干预。

本案例救治以凝血复衡策略为核心，精准指导热射病患者的补凝和抗凝治疗。结合常规凝血检测项目及血栓弹力图结果，本案例主要体现为低纤维蛋白原性及血小板减少性低凝状态，以维持血小板计数在50×10^9^/L和纤维蛋白原1.5 g/L以上为目标进行替代治疗。同时进行抗凝治疗防止凝血底物进一步消耗，阻断DIC的进展。根据热射病性专家共识抗凝药物首选普通肝素，主要考虑普通肝素具有半衰期短、便于监测、一旦过量可用鱼精蛋白中和的优点。故本病例首选普通肝素2 mg/h进行抗凝治疗，并运用血栓弹力图肝素酶对比试验进行精准剂量调控。通过动态监测普通杯R时间与肝素酶杯R时间的比值，实现抗凝强度的实时优化。当比值超出1.5～2的目标范围时，采用灵活的剂量调整策略：比值>2时适当减量，比值<1.5时相应增加给药剂量。每4～6 h进行1次复查，确保抗凝治疗的安全性和有效性。如本例中初始肝素维持剂量为2 mg，6 h后复查血栓弹力图肝素酶对比实验，普通杯中R时间为18.2 min（图1B），肝素酶杯R时间为5.8 min（图1C），计算普通杯R时间与肝素酶杯R时间的比值为2.8大于2，因此需降低肝素剂量，将肝素剂量调至1 mg/h，而后6 h后再复查，直至将普通杯R时间与肝素酶杯R时间的比值调整至1.5～2之间。本案例按照此凝血复衡策略，未出现出血及血栓事件发生。若伴随高出血风险的热射病性凝血病患者，可考虑使用甲磺酸萘莫司他抗凝治疗[3]。

本案例由于早期降温不及时，导致肝功能损害加重，这时热射病性凝血病合并肝衰竭，应进行人工肝治疗。因血源紧张，本案例采用PDF模式治疗，一方面可以清除胆红素和中大分子毒素，还同步补充至少1 600 ml新鲜冰冻血浆，快速改善患者凝血因子活性，成功纠正凝血功能障碍。回顾性临床研究报道经PDF治疗后血管内皮细胞损伤标志物TM水平显著下降，体现PDF治疗可能对血管内皮细胞具有一定保护作用[7]。其核心治疗机制主要包括：清除炎性介质（如IL-6）[8]、抑制凝血系统激活、减轻毛细血管内皮损伤，并通过补充新鲜血浆提高凝血因子水平，全面改善毛细血管内皮细胞功能[9]–[10]。

综上所述，凝血复衡策略联合PDF是快速纠正热射病相关凝血障碍的有效方法。
